# Congenital Afibrinogenemia and Hypofibrinogenemia: Laboratory and Genetic Testing in Rare Bleeding Disorders with Life-Threatening Clinical Manifestations and Challenging Management

**DOI:** 10.3390/diagnostics11112140

**Published:** 2021-11-19

**Authors:** Tomas Simurda, Rosanna Asselta, Jana Zolkova, Monika Brunclikova, Miroslava Dobrotova, Zuzana Kolkova, Dusan Loderer, Ingrid Skornova, Jan Hudecek, Zora Lasabova, Jan Stasko, Peter Kubisz

**Affiliations:** 1 National Center of Hemostasis and Thrombosis, Department of Hematology and Transfusiology, Comenius University in Bratislava, Jessenius Faculty of Medicine in Martin and University Hospital in Martin, 03601 Martin, Slovakia; jana.zolkova@gmail.com (J.Z.); simkovamonika@gmail.com (M.B.); miroslava.dobrotova@gmail.com (M.D.); inkaskornova@gmail.com (I.S.); hudecek@unm.sk (J.H.); jan.stasko@uniba.sk (J.S.); peter.kubisz@uniba.sk (P.K.); 2 Department of Biomedical Sciences, Humanitas University, 20072 Pieve Emanuele, Italy; rosanna.asselta@hunimed.eu; 3 Humanitas Clinical and Research Center IRCCS, 20089 Rozzano, Italy; 4 Biomedical Center Martin, Comenius University in Bratislava, Jessenius Faculty of Medicine in Martin, 03601 Martin, Slovakia; zuzana.snahnicanova@uniba.sk (Z.K.); dusan.loderer@uniba.sk (D.L.); 5 Department of Molecular Biology and Genomics, Comenius University in Bratislava, Jessenius Faculty of Medicine in Martin, 03601 Martin, Slovakia; zora.lasabova@uniba.sk

**Keywords:** afibrinogenemia, hypofibrinogenemia, genetic testing, global coagulation assays, bleeding, thrombosis

## Abstract

Congenital fibrinogen disorders are rare pathologies of the hemostasis, comprising quantitative (afibrinogenemia, hypofibrinogenemia) and qualitative (dysfibrinogenemia and hypodysfibrinogenemia) disorders. The clinical phenotype is highly heterogeneous, being associated with bleeding, thrombosis, or absence of symptoms. Afibrinogenemia and hypofibrinogenemia are the consequence of mutations in the homozygous, heterozygous, or compound heterozygous state in one of three genes encoding the fibrinogen chains, which can affect the synthesis, assembly, intracellular processing, stability, or secretion of fibrinogen. In addition to standard coagulation tests depending on the formation of fibrin, diagnostics also includes global coagulation assays, which are effective in monitoring the management of replacement therapy. Genetic testing is a key point for confirming the clinical diagnosis. The identification of the precise genetic mutations of congenital fibrinogen disorders is of value to permit early testing of other at risk persons and better understand the correlation between clinical phenotype and genotype. Management of patients with afibrinogenemia is particularly challenging since there are no data from evidence-based medicine studies. Fibrinogen concentrate is used to treat bleeding, whereas for the treatment of thrombotic complications, administered low-molecular-weight heparin is most often. This review deals with updated information about afibrinogenemia and hypofibrinogenemia, contributing to the early diagnosis and effective treatment of these disorders.

## 1. Classification and Terminology of Congenital Fibrinogen Disorders

Diseases affecting fibrinogen can be inherited or acquired. Congenital fibrinogen disorders are a heterogeneous group of rare, inherited abnormalities of blood coagulation [1] and can be subclassified in type I and type II disorders. Type I disorders (afibrinogenemia and hypofibrinogenemia) influence the amount of fibrinogen in human blood (fibrinogen level decreased to less than 1.8 g/L), whereas type II (dysfibrinogenemia and hypodysfibrinogenemia) impact primarily the quality of fibrinogen in the circulation [1,2]. Following the laboratory parameters needed for definition of disease seriousness, suggested by the European Network of Rare Bleeding Disorders (EN-RBD) with the support of the International Society of Thrombosis and Hemostasis, quantitative fibrinogen deficiency may be classified into mild hypofibrinogenemia (lower limit of normal level—1.0 g/L), moderate hypofibrinogenemia (0.9–0.5 g/L), severe hypofibrinogenemia (0.5–0.1 g/L), and afibrinogenemia (unmeasurable fibrinogen level <0.1 g/L) [3,4]. The Orphanet classification of rare hematological diseases and the Orphanet classification of rare genetic diseases indicates afibrinogenemia (ORPHA98880) and congenital hypofibrinogenemia (ORPHA101041) [5,6]. According to the Factor XIII and Fibrinogen Subcommittee of the Scientific Standardization Committee of the ISTH, for congenital quantitative fibrinogen disorders, it is necessary to provide an accurate diagnosis and to classify the patients not only based on their fibrinogen levels but also according to their clinical phenotype (Table 1) [1,7].

The first remark on afibrinogenemia was made by Rabe and Salomon in 1920, when the authors discussed an unusual case of 9-year-old boy suffering from repeated bleeding episodes by gastrointestinal bleeding starting shortly after his birthday [4]. Later, Mosesson defined afibrinogenemia as a congenital bleeding disorder of fibrinogen influencing the amount of fibrinogen in human blood; along with other defects of fibrinogen, afibrinogenemia is considered a rare disorder [2,8,9]. Clinical signs of afibrinogenemia and hypofibrinogenemia are various, ranging from being asymptomatic to experiencing dangerous life-threatening bleeding or thromboembolic episodes [1,10,11,12]. In addition, we also know a type of hypofibrinogenemia that is associated with comorbid liver disease, known as hepatic fibrinogen storage disease [13].

## 2. Structure and Function of Fibrinogen

Fibrinogen is a 340-kDa plasma glycoprotein mainly synthesized by liver parenchymal cells [14,15]. The protein is also stored in the platelet alpha granules, thus providing a localized boost in fibrinogen concentration at sites of platelet activation [16]. Fibrinogen is synthetized as a hexamer composed of two copies of three homologous polypeptide chains (Aα, Bβ, and γ), interconnected by a complicated series of disulfide bonds [1,9]. The N-terminus of each chain is disulfide-linked to form the central E region, while the disulfide-linked C terminal of the Bβ, γ, and a portion of the Aα chains form two lateral globular D regions. Fibrinogen polypeptides are encoded by three independent genes, all located on the “p” arm of chromosome 4 (at positions 4q31.3, 4q31.3, and 4q32.1, respectively) [1,17,18]. This region spans approximately 50kb. The gene coding for the fibrinogen Aα chain (*FGA*) has a 7.6-kb size and consists of 6 exons, the Bβ chain gene (*FGB*) presents 8 exons and occupies an 8-kb-long region, and the γ chain gene (*FGG*) encompasses an 8.5-kb region and consists of 10 exons [18,19]. The genes are separately transcribed and translated into polypeptides: 644 amino acids for the Aα chain, 491 amino acids for the Bβ chain, and 437 amino acids for the γ chain [20]. The normal range of fibrinogen is 1.8 to 4.2 g/L in our laboratory. Normal level ranges may vary slightly among different laboratories. Some laboratories use different measurements or different reagents. There may also be slight differences in the normal levels ranges according to gender, age, geographic region, race, or ethnic origin. Fibrinogen has a circulating half-life of 3–5 days [20,21,22,23,24].

The first report identifying fibrinogen was by Dr. Olaf Hammarsten (1841–1932), who elucidated the fact that the precursor of fibrin is fibrinogen, which will clot when exposed to thrombin [2,10]. Fibrinogen serves as a scaffold for platelet aggregation via the activated form of integrin αIIbβ3 (also known as glycoprotein IIb/IIIa). Platelet aggregation via fibrinogen cross-linking provides an initial hemostatic barrier following blood vessel injury as part of the rapid primary hemostatic response. Subsequently, thrombin activation on the platelet surface leads to conversion of fibrinogen to fibrin [25]. The conversion of fibrinogen to fibrin is the final step in the blood coagulation cascade. The important fact is that fibrinogen level is the main determinant of the structure of the resulting fibrin network. Taking everything into account, fibrinogen and fibrin play essential and linking roles in fibrinolytic pathway, cellular interactions, inflammation, preventing microbial invasion and proliferation upon trauma, wound healing, vascular events, and neoplasms [24,26]. On the other hand, fibrinogen also has an anticoagulant role, probably by sequestering unbound thrombin, and participates in the implantation of the fetus [3,27]. During intrauterine development, fibrinogen is present in the blood around the time of termination of hepatic histogenesis and spleen vascularization (10–11 weeks of gestation) with similar levels as in adults [25].

## 3. Epidemiology and Clinical Features

**Figure 1:**
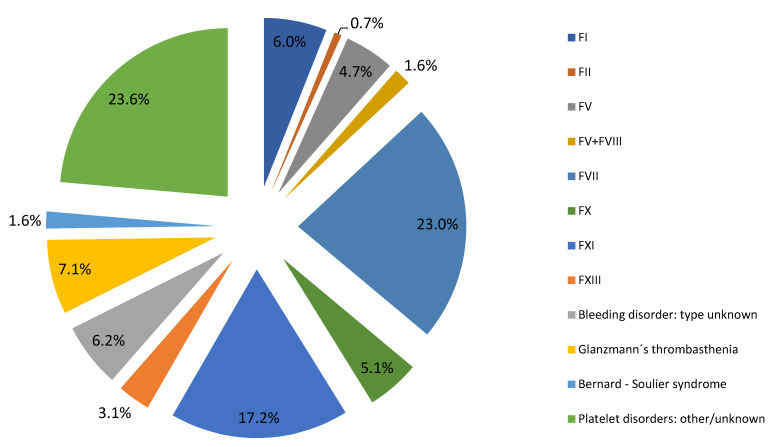
Worldwide distribution of RBDs derived from the WFH survey 2019.

The evaluation of the worldwide rare bleeding disorders prevalence relies on two large surveys that collected epidemiologic data: World Federation of Haemophilia (WFH) and European Network of the Rare Bleeding Disorders (EN-RBD) [28,29]. According to the 2019 World Federation Haemophilia Annual Global Survey, which assessed information from 115 countries, fibrinogen deficiencies represent 0.91% of cases of the total number reported 324,648 people, with bleeding disorders being more prevalent in men when compared with women [30]. Of the total number of rare bleeding disorders (without hemophilia A, hemophilia B, and von Willebrand disease), the worldwide distribution of fibrinogen disorders is 6.0% (Figure 1). However, it should be emphasized that only 76 countries reported the number of patients with fibrinogen deficiencies. This can present a problem in calculating the total world prevalence.

Among European countries, Slovakia, Ireland, and the United Kingdom have the highest prevalence of fibrinogen deficiency 13–18:1,000,000. A key role is played by regular reporting of newly diagnosed cases of rare bleeding disorders in individual national registries. An estimated worldwide prevalence of afibrinogenemia is approximately 1–2 per million in the general population [31,32,33], so it is considered a remarkably rare disorder. The EN-RBD reported 592 patients with congenital fibrinogen deficiency in the database. The prevalence was similar in women (54%) and men (46%) below the age of 60 years, and the prevalence was similar amongst all age groups [34]. According to the latest published cross-sectional international study, quality of life was recorded overall 204 patients with afibrinogenemia from 25 countries, of which more than 50% of patients came from Asia [35]. In populations with frequent consanguineous marriages, the prevalence of afibrinogenemia, like the occurrence of other disorders of hemostasis with autosomal recessive inheritance, is increased [2]. Geographical differences in prevalence reflect high occurrence in children of consanguineous parents in Muslim countries. Comparative data from databases in Iran, Italy, and the United Kingdom points out the worldwide distribution (subjects with plasma fibrinogen ≤10% of normal) [1]. As for congenital hypofibrinogenemia, its prevalence is generally considered to be higher with respect to afibrinogenemia; however, to date, there are no exact data since a large proportion of patients are asymptomatic and hence difficult to be diagnosed.

Apart from the prevalence calculations based on the systematic description of afibrinogenemic/hypofibrinogenemic cases, a complementary approach to determine the worldwide prevalence for these disorders is based on the large amount of genetic data deposited in publicly available databases. In this respect, by a systematic analysis of exome/genome data from approximately 140,000 individuals belonging to the Genome Aggregation Database (GnomAD;), Paraboschi and colleagues showed that the worldwide prevalence for recessively inherited fibrinogen disorders could be 10-fold higher than that so-far described. In addition, it was confirmed that prevalence rates change considerably among populations, going from 1 in 1 million individuals in East Asia, to 24.5 in 1 million people in Europe (excluding non-Finnish Europeans). Importantly, heterozygous individuals could be present in the general population at a frequency of approximately 1 in 100 [36].

**Figure 2:**
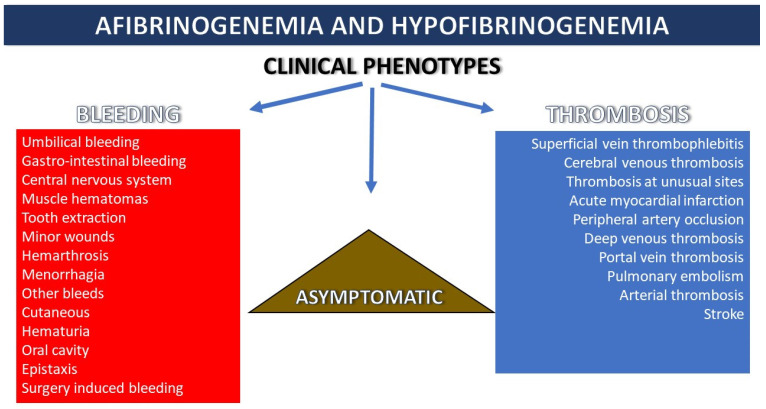
Characterization of clinical phenotype in patients with quantitative fibrinogen disorders.

Clinical manifestations of quantitative fibrinogen disorders are very heterogeneous (Figure 2). In afibrinogenemia, bleeding episodes are the main symptoms, whereas hypofibrinogenemia is more frequently an asymptomatic phenotype. The severity and pattern of clinical manifestations are dependent on the fibrinogen levels [20]. In a retrospective study describing the incidence of bleeding symptoms in 100 patients with afibrinogenemia and hypofibrinogenemia, the mean annual incidence of bleeding episodes per year approached one bleeding per year. This incidence was significantly lower than the incidence of bleeding in hemophilia. Some patients had no bleeding events during the follow-up period [37].

As for bleeding symptoms, umbilical cord bleeding in neonates is generally the first and most frequent sign of the afibrinogenemia, manifesting in 85% of the cases [10]. However, a later age of onset can also occur. The disorder can manifest by uncommon intracranial bleeding in childhood, being the principal cause of death in affected patients. There is also a close relationship with spontaneous splenic rupture in patients with afibrinogenemia [38].

Except these potentially life-threatening complications, the most frequent manifestations of afibrinogenemia are mucosal bleeding, especially menorrhagia, epistaxis, and bleeding in the oral cavity [35,39]. Musculoskeletal bleeding (and also bleeding into the joints) is reported in approximately half of the individuals with afibrinogenemia [1,39,40], and in some studies, it was more prevalent than bleeding from mucosal surfaces. Bleeding from the gastrointestinal and urinary system occurs less frequently [38,41]. First trimester miscarriage and ante- and postpartum hemorrhages were documented, too [2]. Hemoperitoneum is another infrequent gynecologic and obstetric issue of this inherited fibrinogen disorder [2]. Quantitative fibrinogen abnormalities can also lead to complicated wound healing [2,42]. Besides spontaneous bleeding, bleeding after minor injury and excessive bleeding during various interventions are further major manifestations of afibrinogenemia [10]. Last but not least, bleeding symptoms of afibrinogenemic patients are retroperitoneal hemorrhage and hemoptysis [2]. It was documented that patients with low fibrinogen activity have higher occurrence of unprovoked major bleeding events, while patients with sufficient fibrinogen activity were asymptomatic [37].

Paradoxically, patients with afibrinogenemia can experience severe, spontaneous, or repeated thromboembolic complications [1,27,43,44,45,46]. Arterial and also venous thromboembolic episodes in various locations have been reported: thrombosis in peripheral arteries, recurrent myocardial infarction [47], thrombosis of abdominal aorta with peripheral embolization, cerebral [29] or hepatic vein thrombosis [48], or venous thrombosis after the delivery [2,49,50]. In patients with hypofibrinogenemia, diagnosed arterial thrombosis were documented, confirming that thrombosis frequently develops at younger age, it is present in large vessels, its recurrence is not uncommon, and therapeutic management is not clarified yet. Venous thrombosis appears to be the most common thrombotic event [12,19,51,52].

It should be noted that low fibrinogen levels do not compensate a hypercoagulable state [36]. In absence of fibrinogen or at low levels, the small amount of thrombin usually formed remains longer in the circulation, as no or less sequestering on circulating fibrinogen occurs (i.e., antithrombin function of fibrin is impaired) [27]. Besides, thrombin generation has been shown to be increased in the plasma of patients with low levels of fibrinogen [6]. The reasons for increased thrombotic risk are not entirely understood [53]. The pathogenesis at the basis of the paradoxical thrombotic tendency in patients with CFD is likely multifactorial, depending on different exogeneous and endogenous risk factors, such as genetic thrombophilia, use of fibrinogen replacement therapy, immobilization, pregnancy, or trauma [1,12].

## 4. Laboratory Assays

Initial laboratory diagnosis for afibrinogenemia and hypofibrinogenemia should include routine clotting times (prothrombin time (PT), activated partial thromboplastin time (aPTT), or thrombin time (TT)) and fibrinogen assays (activity and antigen) [54]. Fibrinogen activity can be determined by the Clauss or PT-derived fibrinogen assays. The most accurate method to determine fibrinogen level is the Clauss assay, based on the comparison of TT of dilutions of plasma against a plasma standard. PT-derived fibrinogen assay is not a direct determination of fibrinogen activity; measurement derives from the change in light transmission or scatter from a PT curve [22]. Assay for measuring the fibrinogen antigen is performed using luminescent immunoassay (LIA). Immunological assays are based on the determination of antigen concentration using a specific antibody directed against fibrinogen: LIA, EID (immunoprecipitation method called electroimmunodiffusion), or ELISA (enzyme-linked immunosorbent assay) [1,22]. Global hemostasis tests are used in diagnostics in specialized laboratories to better determine the clinical phenotype (Table 2) [2].

**Figure 3:**
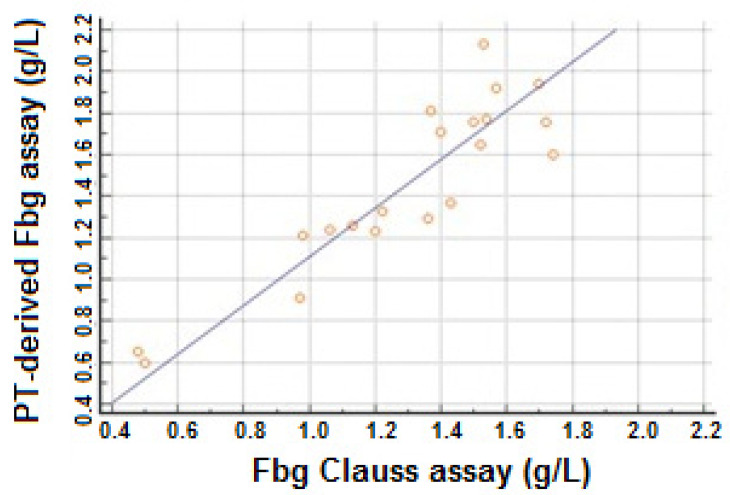
Linear correlation (r = 0.9016; *p* < 0.0001) between PT-derived fibrinogen and fibrinogen Clauss assay in patients with hypofibrinogenemia according to the study published by Skornova et al.

The diagnosis of afibrinogenemia is established on the undetectable level of fibrinogen activity and absence or trace amounts of fibrinogen antigen [55]. All coagulation tests depending on the formation of fibrin as the last step in the coagulation pathway (PT, aPTT, or TT) are infinitely prolonged. Most patients with congenital hypofibrinogenemia are incidentally diagnosed during routine coagulation screening [56]. The diagnosis of hypofibrinogenemia is based on the detection of a proportional reduction of both fibrinogen activity below the lower limit of the normal level (<1.8 g/L) [22]. Routine clotting times are prolonged in proportion to the fibrinogen deficiency. TT is the most sensitive test. In hypofibrinogenemia, there has been confirmed correlation between fibrinogen Clauss assay and PT-derived fibrinogen assay with statistical significance *p* < 0.0001 (Figure 3) [22]. PT-derived fibrinogen assay may have a diagnostic utility. Moreover, we should think that some clinical studies demonstrated that PT-derived level were significantly higher than the Clauss method [57,58].

Peyvandi et al. concluded that in fibrinogen deficiency, there is a strong relationship between coagulation factor activity level and clinical bleeding phenotype [44].

Differential diagnosis between severe hypofibrinogenemia and afibrinogenemia may be difficult due to the limited sensitivity of coagulation tests at fibrinogen levels less than 0.5 g/L [20]. In clinical practice, we use the ratio of functional activity to antigen level [59]. A ratio higher than 0.7 is typical for hypofibrinogenemia, and a ratio lower than 0.7 is typical for dysfibrinogenemia [60]. In addition to routine clotting times, it is necessary to examine the blood count with a focus on the platelet count. Indeed, in 25% patients with afibrinogenemia, mild thrombocytopenia has been reported [61]. Some abnormalities in platelet function tests can be observed: these abnormalities are almost reversible after substitution of fibrinogen, such as platelet adhesion and adenosine diphosphate (ADP)-induced platelet aggregation. On the other hand, thrombin- and collagen-stimulated platelet aggregation is normal [62,63].

Global hemostatic assays, such as thromboelastography (TEG; Haemonetics, Braintree, MA, USA) or rotational thromboelastometry (ROTEM; TEM International, Munich, Germany), in diagnosis of bleeding disorders, prediction of blood transfusion, and mortality in patients with bleeding manifestations has been reviewed [64]. These assays provide us with more information on coagulation efficiency, such as clot formation kinetics, maximum clot strength, and rate of fibrinolysis [56]. ROTEM/TEG are used in acute conditions for rapid assessment of fibrinogen levels. ROTEM/TEG play important roles in diagnosis and transfusion requirements, including fibrinogen replacement in fibrinogen deficiency. Moreover, ROTEM/TEG can be used to detect systemic fibrinolysis (physiologic, hypofibrinolysis, and hyperfibrinolysis) [65,66]. Functional fibrinogen assay using ROTEM (FIBTEM) is an important tool for the assessment of fibrinogen level and its deficiency. FIBTEM assay evaluates the functional stability of fibrin polymerization, the end product of the enzymatic coagulation cascade [67]. The clot strength in FIBTEM is the most used parameter for discrimination of fibrinogen deficiencies and their correlations with fibrinogen levels (Clauss). This strong correlation of FIBTEM results with plasma fibrinogen level confirmed published studies. FIBTEM assay to diagnose fibrinogen deficiency and predict transfusion requirements other variables, such as hematocrits, factor XIII, and fibrinogen concentrate ranges, should be taken into consideration. FIBTEM assay provides data on the external coagulation pathway in which platelets are eliminated. The result of this test provides an estimate of the contribution of fibrinogen to coagulation [68]. FIBTEM assay is sensitive to clot polymerization disorders. Fibrinogen Clauss determines the fibrinogen activity and does not distinguish between qualitative and quantitative defect of fibrinogen. It follows that reduced clot firmness can be differentiated only by performing FIBTEM assay [66].

Point-of-care testing in trauma and surgery with ROTEM/TEG allows us to target the administration of coagulation factors. The FIBTEM assay is used to determine fibrinogen levels and calculate dosage as reported in clinical studies [68]. Maximum clot firmness as one of parameters in FIBTEM is used for the prediction of fibrinogen deficiency and need of fibrinogen concentrate [69]. MCF can confirm the effectiveness and safety of normalization of clot formation after the infusion of fibrinogen in patients with afibrinogenemia and hypofibrinogenemia [70]. Unfortunately, only a few clinical studies focused on predicting the clinical phenotype using ROTEM/TEG [71].

Thrombin generation test is a method which provides a global overview of the hemostatic status. The method evaluates the dynamics of thrombin generation over time [72].

The results of thrombin generation can be examined in patients with afibrinogenemia and hypofibrinogenemia because they can give information about the overall coagulation potential. Thrombin generation could be of particular interest in afibrinogenemia, as it reflects the individual’s overall coagulation potential [73] and can help to identify the patients at the highest risk of thrombotic manifestations. This method tests the overall balance between procoagulant and anticoagulant forces and helps in diagnosis of hypocoagulability and hypercoagulability states [74]. Thrombin generation assay has been used only in the research area. The most recently published study did not show a correlation of the clinical phenotype with thrombin generation levels in CFD. This study has limitations for a low number of cohorts. Further large, multicenter studies are needed to confirm these data [75].

## 5. Genetics of Afibrinogenemia and Hypofibrinogenemia

Afibrinogenemia (Online Mendelian Inheritance in Man, OMIM #202400) Afi is an autosomal recessive disorder, and it is the consequence of mutations in the homozygous or compound heterozygous state in one of the three genes encoding fibrinogen chains. Hypofibrinogenemia (OMIM + 134820, * 134830, and * 134850; dominant trait) has been traditionally considered as a distinct clinical entity from afibrinogenemia; however, it actually represents the phenotypic expression of the heterozygous condition for a single mutation occurring within the fibrinogen gene cluster. For both afibrinogenemia and hypofibrinogenemia, causative mutations can affect the synthesis, assembly, intracellular processing, stability, or secretion of the hexameric fibrinogen leading to decreased levels of circulating fibrinogen [53].

The Human Gene Mutation Database (HGMD) summarizes well the spectrum of mutations located in the *FGA*/*FGB*/*FGG* genes [2,19]. Other relevant sources of information are the LOVD (Leiden Open Variation database) pages dedicated to *FGA*/*FGB*/*FGG* genes (still under construction), which are curated by the European Association for Hemophilia and Allied Disorders (EAHAD), as well as the Human Fibrinogen Database (HFD) curated by the Groupe d’Etude sur l’Hemostase et la Thrombose. It is important to underline that all these sources of information indeed report on mutations in the fibrinogen cluster associated not only with afibrinogenemia and hypofibrinogenemia but also with other fibrinogen disorders (i.e., hypo-dysfibrinogenemia, dysfibrinogenemia, fibrinogen storage disease, and hereditary renal amyloidosis) [36].

By consulting the public version of the HGMD repository (accessed on 20 July 2021), the extreme allelic heterogeneity of congenital fibrinogen disorders is evident: a total of 363 mutations have been found in the fibrinogen gene cluster, of which 142 are in the *FGA* gene (39%), 90 in *FGB* (25%), and 131 in *FGG* (36%). These numbers are even higher when accessing the restricted section of this database (169, 107, and 153 genetic variants described in the *FGA*, *FGB*, and *FGG* genes, respectively).

The distribution of fibrinogen-related mutations, according to their type, is summarized in Table 3 and Table 4. Here, it is possible to appreciate from one side the high prevalence of missense mutations (especially in the *FGG* gene, where they reach the highest level, i.e., 76% of the total) and, on the other side, the low frequency of gross deletions/duplications/rearrangements (i.e., only nine pathogenic variants have been described collectively for the three genes).

The distribution of fibrinogen-related mutations according to the associated phenotype is reported in Table 4. From the table, it clearly emerges the great variety of phenotypes associated with variants in the fibrinogen genes; on the other hand, this also presents a difficulty for physicians to classify patients on the basis of their fibrinogen levels rather than of their symptoms. For instance, among the 93 listed afibrinogenemia-causing mutations, carrier patients were classified on the basis of plasma fibrinogen, but among them, it is possible to find those having experienced bleeding episodes, those that were asymptomatic, or even those with thrombotic phenotypes.

Notwithstanding this plethora of phenotypes, the great majority of mutations described in the fibrinogen cluster have been associated with “genuine” afibrinogenemia (93 mutations) or hypofibrinogenemia (84 mutations; Table 4). An additional 12 mutations can be easily reconducted to type I quantitative fibrinogen disorders (i.e., two associated with “Decreased fibrinogen levels?”, one with “Decreased fibrinogen levels”, three with “Afibrinogenaemia?”, three with “Afibrinogenaemia/hypofibrinogenemia”, one with “Afibrinogenemia with recurrent venous thromboembolism”, and two with “Hypofibrinogenaemia?”; see phenotypes listed in Table 4). Overall, since the description of the first afibrinogenemia-causing mutation 22 years ago (an 11-kb deletion affecting the *FGA gene)*, ≈200 different pathogenic variants have been reported [76]. The majority of mutations leading to afibrinogenemia are located in *FGA* (55 out of 93, ≈60%), whereas hypofibrinogenemia-causing variants cluster in the *FGG* gene (41 out of 84, ≈49%; Table 4). It is also interesting to underline that the mutational spectrum of afibrinogenemia is characterized by the presence of at least two recurrent mutations, both located in *FGA* and both typical of Caucasian patients: the already mentioned 11-kb deletion and the c.510 + 1G > T splice site mutation; in a cohort of 74 unrelated probands, Casini and colleagues highlighted that these variants, respectively, represent 12.2 and 23.6% of the mutated alleles [77].

## 6. Genetic Diagnosis and Antenatal Diagnosis

DNA Sanger sequencing of the coding portions of *FGA*/*FGB*/*FGG* has been the gold standard in the last 20 years for the identification of molecular defects underlying afibrinogenemia and hypofibrinogenemia. This is due to the fact that these disorders show extreme allelic heterogeneity (with most mutations being “private” defects and only few being recurrent). Hence, genetic screenings have traditionally been performed by polymerase chain reaction (PCR) amplifications of exons, splice sites, and promoter regions of the *FGA*/*FGB*/*FGG* genes, followed by direct sequencing of the amplified products. This approach has been coupled in some laboratories with a screening based on the Multiplex Ligation Probe Amplification (MLPA) method to search essentially for large deletions otherwise missed by Sanger sequencing.

The molecular screening based on Sanger sequencing has, of course, its pros and cons. In fact, despite its standardization, the ease of execution, and the relative ease of analyzing the results, Sanger sequencing can sometimes be costly, time consuming, and quite complicated (like in the case of large and multi-exonic genes; in the case of fibrinogen, there is the necessity to screen the entire cluster). In addition, it is possible to miss mutations lying deep in introns unless the entire gene is sequenced.

The advent of next-generation sequencing (NGS) techniques is, of course, changing the overall picture of genetic diagnosis, and “inherited bleeding, thrombotic, and platelet disorders” (collectively called BPDs and including a- and hypo-fibrinogenemia), are not exceptions. In this frame, a first seminal paper appeared in the literature in 2016: Simeoni and colleagues [78] developed a high-throughput sequencing platform targeting a total of 63 genes (the ThromboGenomics platform) and applied their design to 137 individuals with a suspect of BPD. The diagnostic yield was 46%, underlying that there is still a need for identifying novel molecular causes of BPDs. Similar results were later obtained by Downes et al. [79], who applied the same platformto screen 2396 BPD patients: they reached a diagnostic yield of 50%, identified hundreds of mutations (half of which novel), and also proposed an oligogenic model of inheritance for some patients.

Specifically concerning fibrinogen disorders, the largest study based on NGS screening of affected patients appeared in 2019 [80]. Here, a total of 17 Spanish patients (suffering from a-, hypo, or dys-fibrinogenemia) were screened by using a NGS approach based on sequencing the complete *FGA*, *FGB* and *FGG* genes (i.e., also including introns). All patients were associated with one/two mutations, thus underlying the overall good performance of the adopted strategy [80].

For the future, it is conceivable that—also thanks to the significant drop in costs—genetic diagnosis will be carried out by using more comprehensive NGS strategies, i.e., whole-exome sequencing (WES) or whole-genome sequencing (WGS). These approaches will offer novel intriguing possibilities: (i) to highlight the oligogenic nature of specific conditions/patients; (ii) as for WGS, to identify “elusive” mutations (large rearrangements, large deletions, deep-intonic mutations, mutations in enhancers, or other regulatory regions); and (iii) to identify modulators of the phenotype. In this last case, it could be possible to answer to open questions, such as the differences in the severity of hemorrhagic manifestations in individuals carrying the same mutation or the reason why some afibrinogenemic patients are susceptible to develop thrombosis. Of course, we are aware that these are currently speculations (and hopes). However, it should be noted that some encouraging examples related to congenital fibrinogen disorders come from the literature. For instance, dysfibrinogenemias are often related to pathogenic variants affecting residues p.Gly17, p.Pro18, p.Arg19, and p.Val20 in the amino-terminal region of the fibrinogen Aα-chain. However, while mutations at residues p.Gly17, p.Pro18, and p.Val20 are exclusively linked to bleeding tendency, the clinical phenotype of patients with mutations at amino acid p.Arg19 can vary from bleeding to thrombotic tendency [81]. In this frame, the case described by Bor and colleagues [81] is intriguing: they exome sequenced a Danish family with thrombotic episodes, revealing from one side the dysfibrinogenemia-causing mutation Aα-p.Arg19Gly and from the other a series of single-nucleotide polymorphisms located in *FGA*, *FGB*, and *FGG*. These polymorphisms are possibly responsible for an increased fibrin fiber thickness and fibrin mass-to-length ratio, thus suggesting that the combination of genotypes may contribute to the thrombogenic phenotype of these patients

A final thought concerns the management of recessively inherited coagulation disorders, which depends on two fundamental steps: genetic counseling in consanguineous marriages and prenatal diagnosis in families at risk for having members with severe form of the disorder. These approaches are not easily realizable in the praxis [31].

Pregnant women with a family history, predominantly those with a history of consanguinity, ought to be properly counseled with regard to risk of having a child with the disorder. If we know the mutation, prenatal analysis could be planned: in fact, for a disease such as afibrinogenemia, where bleeding after loss of the umbilical cord stump is frequent and, in some cases, lethal, the prenatal diagnosis of an affected infant can allow treatment immediately after birth and before the first bleeding manifestation. However, the issue of prenatal diagnosis in rare bleeding disorders is still under debate [82], especially considering that, in most cases, it is performed using invasive procedures (such as withdrawal of chorionic villi) that can have dramatic consequences on the fetus. The first prenatal diagnosis for afibrinogenemia was done for a Palestinian family with two affected daughters by Neerman-Arbez et al. in 2003 [83]. In babies of known/suspected carrier couples, cord blood detection of genetic mutations can be done. Indirect prenatal testing by the use of linkage analyses might be an option in rare inherited bleeding disorders, too [82].

## 7. Treatment

Substitution therapy is effective in the treatment of bleeding episodes in congenital fibrinogen disorders [55,75]. If possible, specific plasma-derived factor concentrate deprived of active viruses ought to be administered preferentially in rare bleeding diseases. Fresh frozen plasma (FFP) or cryoprecipitate should be administered in the unavailability of plasma-derived factor concentrate [3]. FFP has several disadvantages: the decreased amount of fibrinogen that is infused leads to the need to repeat the administration more times to achieve the appropriate fibrinogen level; in addition, there are transfusion-related risks (e.g., transfusion-related acute lung injury (TRALI) and transmission of virus infections) [75,76]. In addition, cryoprecipitate has many of these handicaps; it also needs compatibility testing, thawing, and it has a complicated application [76]. It is not provided in many Western European countries, but it is still administered in the United States and the United Kingdom [66]. A standard dose of 10–20 units (500–1000 mL) of methylene blue-cryoprecipitate is awaited to raise fibrinogen activity by 0.6–1.2 g/L in a 70 kg adult [84,85]. It may continue daily or every other day in the absence of consumption with frequent monitoring of the activity, according to the indication and reaction [76].

To date, we know six fibrinogen concentrates: RiaSTAP /Haemocomplettan^®^ P (CSL Behring, Marburg, Germany), Fibryga^®^ (Octapharma, Lachen, Switzerland), FibCLOT^®^/CLOTTAFACT^®^ (LFB, Les Ullis, France), Fibrinogen HT^®^ (Benesis, Osaka, Japan), FibroRAAS^®^ (Shangai RAAS, Shangai, China), and FIB Grifols^®^ (Grifols, Barcelona, Spain) [2,10,37,69,86,87].

Fibrinogen replacement treatment, especially the most frequently administered concentrate Haemocomplettan P/Riastap, is suggested as therapy for spontaneous bleeding events and as prophylaxis before surgical interventions or against unprovoked bleeds in individuals with congenital and acquired fibrinogen deficiency [56,62].

**Figure 4:**
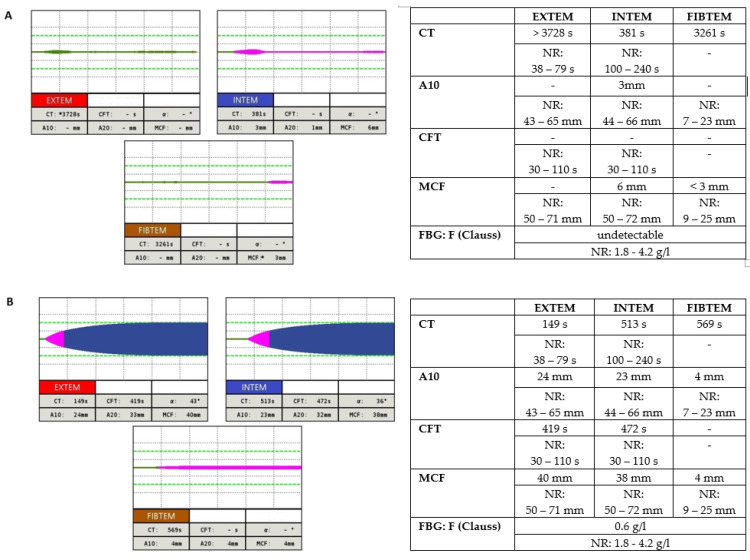
Rotational thromboelastometry (EXTEM, INTEM, FIBTEM) results and fibrinogen activity in the patient with afibrinogenemia from our center. (**A**) Basal level; (**B**) 30 min after administration of 24 mg/kg fibrinogen concentrate (Haemocomplettan^®^ P.

Safety (less frequent allergic reactions), accuracy, simple dosing in small amounts, and rapidity of administration are the primary reasons for the paramount use of fibrinogen concentrates [62,87]. On the other hand, venous or arterial thrombotic events were present in 30% of subjects with fibrinogen deficiency treated by fibrinogen concentrates, mostly in afibrinogenemics [3,63]. Moreover, the potential risk of prion-transmission or venous access complications are other side effects of their usage [2]. As the pharmacokinetic properties of fibrinogen after substitution show a large among-patients variability, tailoring of the prophylactic regimen to the pharmacokinetics of the individuals can be a possibility [87]. This is the reason why the individualized management is considered to be “a job of mastery” (Figure 4) [2]. 

According to the pharmacokinetics of fibrinogen described above, a standard dosage of fibrinogen concentrate of 4–6 g should raise plasma fibrinogen activity by 1.0–1.5 g/L in a 70 kg adult [3,63]. For a better calculation of the dosage, the following formula may be helpful: dose (g) = awaited increase in g/L x plasma volume. Plasma volume calculate: 0.07 × (1 − hematocrit) × weight (kg) [55]. Moreover, the fibrinogen dose using the FIBTEM assay can be calculated as follows: Fibrinogen concentrate dose (g) = (target FIBTEM MCF (mm) − actual FIBTEM MCF (mm)) × (body weight (kg)/70) × 0.5 g/mm [68].

For unprovoked hemorrhage, suggested fibrinogen concentrations are >1 g/L until hemostasis is normalized and >0.5 g/L until the bleeding surface is entirely restored [55]. Fibrinogen concentrate of 50–100 mg/kg every 2–4 days with resultant fibrinogen activity >1.0–1.5 g/L was normally needed to treat or prevent spontaneous or surgical hemorrhage [37,63]. Therefore, for these situations in afibrinogenemia, hypofibrinogenemia, or hemorrhagic dysfibrinogenemia, the United Kingdom Hemophilia Centre Doctors’ Organization guidelines suggest assessment of fibrinogen concentrate in the dose 50–100 mg/kg, with smaller doses repeated if needed at 2–4 day intervals to maintain fibrinogen activity >1.0 g/L [3].

Antifibrinolytics can be helpful in the cases of mucosal bleeding, but they ought to be used very carefully in subjects with a personal or family history of thrombotic episode. Estrogen/progesterone derivatives have been given in menorrhagia [3,76].

Moreover, in prevention, the trough fibrinogen level to be targeted is unknown because fibrinogen concentrate has been potentially linked to a risk of thrombosis.

For women with fibrinogen activity <0.5 g/L or with previous poor pregnancy outcomes, the prophylaxis during pregnancy with fibrinogen concentrate at firstly 50–100 mg/kg twice per week, tailored to retain trough fibrinogen activity >1 g/L is suggested [3,76].

### Management of Thrombotic Complications

The management of thrombotic complications in patients with afibrinogenemia and hypofibrinogenemia is problematic because of their bleeding tendency. Antithrombotic treatment should be individualized and the potential risk of thrombosis weighed against the likely benefits of treatment. An accurate thromboprophylaxis with low molecular weight heparin should be considered in all patients with thrombotic history [6]. Some authors recommend use of compression stockings and low molecular weight heparin in patients with a history of thrombosis that undergo surgery [12]. In patients who develop thrombotic complications following replacement therapy, some authors continue the latter if indicated and co-administer low-molecular-weight or unfractionated heparin [5]. A case study also described the successful use of a new oral anticoagulant (rivaroxaban) for the anticoagulant management in patient with afibrinogenemia and severe hypofibrinogenemia [12,88]. The treatment of thrombotic episodes is very demanding due to the high risk of bleeding [5,16]. Further studies are required to determine the optimal postoperative thromboprophylaxis in CFD.

## 8. Conclusions

Congenital afibrinogenemia and hypofibrinogenemia are rare bleeding diseases. Even though the number of patients studied is quite significant, research in this area, performed in the clinics and laboratories, is still very important [2]. In fact, despite the current progresses, many issues remain, especially regarding the correct way to achieve more effective treatment as well as regarding the set-up of optimal schedule for the prophylactic management of the affected patients [89]. In addition, individuals with afibrinogenemia and hypofibrinogenemia may experience severe life-threatening bleeding or thromboembolic episodes [51]. A better understanding of pathogenic mechanisms as well as the setting up of new/ameliorated diagnostic procedures also at genetic level are therefore an urgent need for the multidisciplinary long-term management of these patients.

## Figures and Tables

**Table 1:** Classification of congenital quantitative fibrinogen disorders [7].

Type and Subtypes Quantitative Fibrinogen Disorders	Descriptions
**Afibrinogenemia**	
**A**. Afibrinogenemia	Afibrinogenemia and bleeding phenotype or asymptomatic individuals
**B**. Afibrinogenemia with a thrombotic phenotype	Afibrinogenemia and thrombotic phenotype
**Hypofibrinogenemia**	
**A**. Severe hypofibrinogenemia	Functional fibrinogen level ˂0.5 g/L
**B**. Moderate hypofibrinogenemia	Functional fibrinogen level between 0.5–0.9 g/L
**C**. Mild hypofibrinogenemia	Functional fibrinogen level between 1.0 g/L and lower limit of normal level
**D**. Hypofibrinogenemia with fibrinogen storage disease	Congenital hypofibrinogenemia with histologically proven accumulation of fibrin in hepatocytes

**Table 2:** Diagnostic procedures of quantitative fibrinogen disorders.

Diagnostic Procedures	Afibrinogenemia	Hypofibrinogenemia
Prothrombin time (PT)	extremely prolonged	prolonged/normal—depending on fibrinogen levels
Activated partial thromboplastin time (aPTT)	extremely prolonged	prolonged/normal—depending on fibrinogen levels
Thrombin time (TT)	extremely prolonged	prolonged/normal—depending on fibrinogen levels
Reptilase time (RT)	extremely prolonged	prolonged/normal—depending on fibrinogen levels
Fibrinogen activity (FBG: F (Clauss))	undetectable	proportional decrease
Fibrinogen antigen (FBG: Ag)	undetectable	proportional decrease
PT-derived fibrinogen assay	undetectable	decrease, proportional to fibrinogen levels
Genotype (*FGA*, *FGB*, *FGG* genes)		
Global hemostasis tests (research laboratories)		

**Table 3:** Mutational spectra of congenital fibrinogen disorders in the *FGA*, *FGB*, and *FGG* genes.

	Number of Mutations		
Mutation Type	*FGA*	*FGB*	*FGG*
Missense	54	55	100
Nonsense	25	12	4
Splicing	11	7	9
Regulatory	3	3	1
Small deletions	28	8	15
Small insertions	11	2	0
Small indels	4	1	1
Gross deletions	5	1	1
Gross insertions/duplications	1	0	0
Complex rearrangements	0	1	0
**Total (public HGMD repository)**	**142**	**90**	**131**
	(169)	(107)	(153)

Data were retrieved from the Human Gene Mutation Database (HGMD) (http://www.hgmd.cf.ac.uk/, accessed on 20 July 2021). In parentheses, the number of mutations available through the restricted access to the database.

**Table 4:** Distribution of mutations in the *FGA*, *FGB*, and *FGG* genes according to the associated phenotype.

	Number of Mutations		
Disease/Phenotype	*FGA*	*FGB*	*FGG*
Afibrinogenemia	55	24	14
Dysfibrinogenemia	38	17	50
Renal amyloidosis	14	0	0
Hypofibrinogenemia	13	30	41
Fibrinogen variant	4	3	7
Susceptibility to venous thromboembolism	3	0	0
Decreased fibrinogen levels?	2	0	0
Decreased fibrinogen levels	0	0	1
Hypodysfibrinogenemia	2	3	9
Afibrinogenaemia?	1	2	0
Afibrinogenemia/hypofibrinogenemia	1	1	1
Afibrinogenemia with recurrent venous thromboembolism	1	0	0
Amyloidosis, Ostertag-type	1	0	0
Deep vein thrombosis?	1	0	0
Dysfibrinogenemia?	1	0	1
Hemorrhages	1	3	0
Association with increased post-stroke mortality	1	0	0
Menorrhagia	1	0	1
Thrombosis	1	0	0
Venous thromboembolism?	1	0	0
Association with cerebral infarction	0	1	0
Epistaxis	0	1	1
Hypofibrinogenaemia?	0	1	1
Association with increased clot stiffness	0	1	0
Increased plasma fibrinogen levels	0	1	0
Thrombotic tendency	0	1	0
Protection against venous thromboembolism	0	1	0
Increased risk for deep venous thrombosis	0	0	1
Hypofibrinogenaemia with hepatic storage	0	0	3
Total (public HGMD repository)	142	90	131

Data were retrieved from the HGMD (http://www.hgmd.cf.ac.uk/, accessed on 20 July 2021) and references therein. Phenotypes are all those reported in such database (the question mark the uncertainty of the associated diagnosis).
